# Design and Application of Fluorescence Probes for Gold Nanocage Complex Perovskite Quantum Dots

**DOI:** 10.3390/nano16030168

**Published:** 2026-01-26

**Authors:** Ying Liu, Yinglian Wu, Hongliang Zhang, Ruiqi Bao, Jingjing Wang, Wei Chen

**Affiliations:** 1 Xuzhou College of Industrial Technology, Xuzhou 221140, China; liuying70@126.com; 2 Jiangsu Key Laboratory of Advanced Laser Materials and Devices, School of Physics and Electronic Engineering, Jiangsu Normal University, Xuzhou 221116, China; 18260728693@139.com (Y.W.); 2020231368@jsnu.edu.cn (H.Z.); 2020241403@jsnu.edu.cn (R.B.); 3 School of Chips, XJTLU Entrepreneur College (Taicang), Xi’an Jiaotong-Liverpool University, Suzhou 215400, China

**Keywords:** gold nanocages, perovskite quantum dots, fluorescence probes, fluorescence resonance energy transfer effect

## Abstract

In this study, a gold nanocage composite perovskite quantum dot fluorescent probe (MB-GNCs-PQDs) was designed and constructed. The GNCs-PQDs composite system was formed by the combination of gold nanocages (GNCs) and perovskite quantum dots (PQDs). Spectral analysis confirmed that its fluorescence intensity was significantly enhanced by 15.38% compared with that of pure PQDs. Furthermore, amino modification was performed on the nanomaterial. Through the specific design of molecular beacons (MB), the fluorescence emission spectrum of the probe was matched with the absorption peak of the quencher group BHQ2, and the effective closure of the fluorescence signal was achieved based on the Fluorescence Resonance Energy Transfer (FRET) effect. Subsequently, MB was immobilized on the surface of the composite system via amino covalent conjugation to complete the probe preparation. The prepared probe was applied to the detection of miRNA-4529-3P and miR-301b-3p, which are tumor markers of non-small cell lung cancer (NSCLC). The hybridization of target molecules with MB could trigger the disruption of FRET and the recovery of fluorescence signal, exhibiting excellent recognition performance. This study provides an experimental basis for the preparation of composite fluorescent probes, and the developed probe has potential application value in the field of tumor marker detection.

## 1. Introduction

As an analytical tool capable of converting molecular recognition events into detectable fluorescent signals, fluorescent probes occupy an irreplaceable position in modern scientific research and practical applications [1,2]. In the biomedical field, they provide visualization approaches for the early diagnosis of diseases, cell imaging, and drug screening [3]; in environmental monitoring, they can rapidly detect pollutants such as heavy metal ions and pesticide residues in water and soil, offering timely data support for ecological environmental protection [4]; and in food safety detection, they are able to efficiently identify pathogenic bacteria, toxins, and illegal additives in food, thus safeguarding public dietary safety [5]. These extensive application scenarios impose stringent requirements on the performance of fluorescent probes. An ideal fluorescent probe should simultaneously possess core characteristics, including good photostability and excellent biocompatibility [6].

Gold nanocages (GNCs), as a novel type of nanomaterial, have attracted significant attention due to their unique hollow structure and localized surface plasmon resonance (LSPR) effect [7,8]. The LSPR effect can enhance the radiative transition of surrounding fluorescent molecules, remarkably boosting the fluorescence intensity and demonstrating great potential in the field of biosensing [9,10,11]. Perovskite quantum dots (PQDs), characterized by their high fluorescence quantum yield, narrow emission spectrum, and tunable luminescent properties, are ideal candidate materials for fluorescent probes, capable of providing efficient signal output [12,13]. The combination of PQDs and GNCs not only integrates the advantages of both but also further optimizes the optical properties through the synergistic effect between nanostructures, offering a new strategy for the construction of high-performance fluorescent probes [14].

Fluorescence resonance energy transfer (FRET) technology is one of the important mechanisms in the design of fluorescent probes [15,16]. By rationally designing the spectral matching of donor fluorophores and acceptor quenching groups, the “on-off” regulation of fluorescence signals can be achieved, effectively reducing background interference and enhancing detection sensitivity [17]. Molecular beacons (MBs), as single-stranded nucleic acid probes, can undergo conformational changes after specific hybridization with target molecules, triggering the activation or deactivation of the FRET effect, and are thus widely used in the detection of nucleic acid-based tumor markers [18,19].

Although existing studies have separately confirmed the LSPR fluorescence enhancement effect of GNCs, the excellent luminescent properties of PQDs, and the specific detection advantages of the FRET-MB system, the development of current fluorescent probes still faces key challenges: most probes only focus on the optimization of a single performance, and it is difficult to effectively integrate high fluorescence enhancement efficiency, low background signal (high signal-to-noise ratio, SNR), and potential multifunctionality (e.g., photothermal properties) into a single system [20]. This limitation leads to the fact that existing probes often fail to meet the requirements of high-sensitivity and high-selectivity detection when detecting low-abundance miRNAs in complex biological samples, due to insufficient fluorescent signal intensity, obvious background interference, or single functionality.

On the basis of the aforementioned research status, this paper aims to design and construct a novel multifunctional composite fluorescent probe with high sensitivity and high specificity. A GNCs-PQDs system was formed by combining perovskite quantum dots with gold nanocages, and the LSPR effect of GNCs was used to significantly enhance the fluorescence intensity of PQDs. Meanwhile, the specific recognition of molecular beacons (MB) was combined with FRET technology to achieve accurate off-on regulation of fluorescent signals, effectively reducing background interference. In addition, the inherent photothermal properties of GNCs were retained, endowing the probe with potential multifunctional application prospects. The prepared probe was applied to the detection of miRNA-4529-3P and miR-301b-3p, which are tumor markers of non-small cell lung cancer (NSCLC), aiming to address the problems of insufficient sensitivity and selectivity in the detection of low-abundance tumor markers. This study provides an experimental basis for the preparation of multifunctional composite fluorescent probes, and it has important potential application value in the field of tumor marker detection.

## 2. Experimental **Preparation**

### 2.1. Instruments and Reagents

T-125 Intelligent Temperature Controller (Shenzhen Topower Electronic Technology Co., Ltd., Shenzhen, China); D2012 Mini Centrifuge (Thermo Fisher Scientific, Waltham, MA, USA); Eppendorf Pipette (Dragon Lab Instruments Co., Ltd., Beijing, China); ZNCL-TS-250 mL Magnetic Stirring Heating Mantle (Shanghai Anchor Instrument Co., Ltd., Shanghai, China); JJ 300 Precision Electronic Analytical Balance (Mettler Toledo Instrument Co., Ltd., Zurich, Switzerland); High-Purity Nitrogen Gas (Xuzhou Special Gas Co., Ltd., Xuzhou, China); Haier Refrigerator (Haier Group, Qingdao, China); GI-54DWS High-Pressure Sterilization Autoclave (Shanghai Danding International Trading Co., Ltd., Shanghai, China).

Cesium carbonate (Cs_2_CO_3_), lead bromide (PbBr_2_), 1-octadecene (ODE), oleylamine (OAM), and HAuCl_4_ were purchased from Shanghai Aladdin Biochemical Technology Co., Ltd. (Shanghai, China). Lead bromide (PbBr_2_), cyclohexane (C_6_H_12_), oleic acid (OA), 1-ethyl-3-(3-dimethylaminopropyl) carbodiimide (EDC), and PBS buffer were obtained from Sinopharm Chemical Reagent Co., Ltd. (Shanghai, China). Silver nitrate, sodium citrate dihydrate, chloroauric acid, sulfo-NHS, anhydrous ethanol, and PVP were sourced from Shanghai Huayuan World Trade Co., Ltd. (Shanghai, China). Hydrochloride (EDC) and *N*-hydroxysuccinimide (NHS) were acquired from Shanghai Macklin Biochemical Technology Co., Ltd. (Shanghai, China), MB, miRNA, and hydrochloric acid (HCl) were procured from Shanghai Sangon Biotech Co., Ltd. (Shanghai, China).

### 2.2. Preparation of PQDs

In a three-neck flask under nitrogen protection, 0.203 g of cesium carbonate was mixed with 10 mL of octadecene and 1 mL of oleic acid. The mixture was sequentially degassed at 120 °C for 10 min and magnetically stirred at 150 °C for 60 min, followed by cooling to obtain the cesium precursor solution. Subsequently, 0.188 mmol of lead bromide and 5 mL of octadecene were deoxygenated at 120 °C for 30 min, followed by the sequential addition of 1.5 mL oleic acid and oleylamine. The temperature was raised to 160 °C, and 0.4 mL of the cesium precursor solution was rapidly injected. The reaction was quenched in an ice bath within 3–5 s, and CsPbBr_3_ perovskite quantum dots (PQDs) were obtained through hexane dispersion and centrifugation.

### 2.3. The Preparation of GNCs

Ten milliliters of polyvinylpyrrolidone (PVP) solution with a concentration of 4 mg/mL was added to a three-necked flask, followed by the addition of 1 mL of silver nanoparticle templates. The silver nanoparticles used were silver nanocubes with an edge length of approximately 50 nm, synthesized via the polyol method. Their concentration was calibrated by the absorbance (optical density, OD value) of their ultraviolet-visible (UV-Vis) absorption spectrum in deionized water at 400 nm, which was approximately 1.0. Subsequently, the three-necked flask was put on a magnetic stirrer for heating, with the heating temperature set at 100 °C and the stirring speed at 500 r/min. After heating for 15 min, chloroauric acid (HAuCl_4_) solution was added at a constant rate of 3 mL/min. Upon the completion of the addition, the reaction system was continuously stirred at 100 °C and 500 r/min until the color of the solution changed from grayish green to a stable blue-green (which took approximately 15–20 min), indicating the completion of the etching-displacement reaction.

After the reaction, the system was cooled down to 25 °C for purification. Specifically, an excess of saturated sodium chloride (NaCl) solution was first added to aggregate and remove residual PVP and reaction by-products. Then, the mixture was centrifuged at 8000 rpm for 10 min to collect the precipitate. The obtained precipitate was resuspended in deionized water and centrifuged again under the conditions of 8000 rpm for 5 min. This washing process was repeated twice. Finally, the purified product was redispersed in deionized water and stored at 4 °C.

### 2.4. Preparation of GNCs-PQDs

The surface-functionalized composite of gold nanocages (GNCs) and quantum dots (QDs) was achieved in phosphate-buffered saline (PBS) buffer via the sulfo-NHS/EDC coupling system. Finally, the GNCs-PQDs composite nanomaterial was obtained by centrifugation. The detailed procedure was as follows: 1 mL of the aqueous GNCs solution (with a concentration of approximately 0.05 mg/mL) was mixed with 0.1 mL of the cyclohexane solution of PQDs (with a concentration of approximately 10 μM), followed by vortex mixing to achieve initial contact. This ratio was optimized through preliminary experiments, aiming to ensure a sufficient PQD loading capacity while avoiding excessive aggregation. Subsequently, 1 mL of PBS buffer (pH 7.4) was added to the above mixture. Thereafter, freshly prepared aqueous solutions of 1-ethyl-3-(3-dimethylaminopropyl)carbodiimide hydrochloride (EDC·HCl) and sulfo-N-hydroxysulfosuccinimide (sulfo-NHS) were added sequentially, with their final concentrations adjusted to 5 mM and 10 mM, respectively. At room temperature (25 °C), the reaction mixture was gently shaken in a shaker at 300 rpm for 2 h.

After the reaction was completed, the mixture was centrifuged at 12,000 rpm for 15 min to remove the supernatant. The collected GNCs-PQDs were washed once with PBS buffer and finally resuspended in 1 mL of PBS for subsequent use. In this step, EDC was used to activate the carboxyl groups potentially present on the surface of GNCs or on the ligands of PQDs, thereby facilitating the formation of amide bonds between the two components.

### 2.5. Preparation of Fluorescent Probe MB-GNCs-PQDs

The amino-terminated GNCs-PQDs nanomaterials were ultrasonically dispersed in ethanol solution (1 mL), followed by the addition of 5 mg of carbodiimide (EDC) and *N*-hydroxysuccinimide (NHS). After shaking and dissolution, the pH of the system was adjusted to 6.5 with 2 μM hydrochloric acid to achieve the amino activation. In total, 20 μL of the carboxylated molecular beacon (MB) solution (1 μM) was mixed with 50 μL of the activated nanomaterial suspension, and the mixture was shaken in the cross-linking reaction system for 30 min to achieve the directional coupling of MB. The reaction product was centrifuged at 10,000 rpm for 5 min twice to remove the free MB, and the final MB-GNCs-PQDs composite probe was stored at 4 °C for future use.

### 2.6. Instrument Characterization

The morphology and structure were characterized by transmission electron microscopy (FEI TECNAI G2 F20, FEI Company, Hillsboro, OR, USA). The composition of the material was tested by X-ray diffraction. The optical properties were determined by a fluorescence spectrophotometer (F-4600, Hitachi High-Tech Corporation, Tokyo, Japan), a transient absorption spectrometer, and an ultraviolet-visible-near-infrared spectrophotometer (Lambda 950, PerkinElmer Inc., Boston, MA, USA).

## 3. Experimental Results and Discussion

### 3.1. The Basic Theories of LSPR and FRET

The integration of localized surface plasmon resonance (LSPR) and fluorescence resonance energy transfer (FRET) represents a significant research direction in the field of nanophotonics, with their synergistic effects demonstrating unique advantages for future applications. When metallic nanostructures are excited by light of a specific wavelength, the collective oscillation of free electrons on their surface occurs under the influence of the alternating electromagnetic field—a phenomenon known as localized surface plasmon resonance (LSPR). Driven by the electric field of incident light, the electron cloud shifts away from the atomic nucleus and subsequently undergoes periodic oscillations due to the Coulomb restoring force, resulting in a locally enhanced electromagnetic field on the nanoparticle surface.

The physical mechanism of LSPR arises from the unique interaction between metallic nanostructures and light, characterized by a strong resonance response when the frequency of incident light matches the natural oscillation frequency of the free electrons within the nanostructure. Under the alternating electric field of light, free electrons in metallic nanoparticles undergo collective displacement relative to the positively charged ion lattice. The resulting charge separation induces a Coulomb restoring force that seeks to return the electron cloud to its equilibrium position, forming a system analogous to a driven damped harmonic oscillator (with the driving force from the light’s electric field, restoring force from Coulomb interaction, and damping arising from electron collisions). At resonance, when the light frequency aligns with the system’s eigenfrequency, the oscillation amplitude of the electron cloud is maximized, significantly enhancing the absorption and scattering of light by the nanoparticle and generating a highly localized electromagnetic field, particularly near regions of high curvature.

The resonance behavior is highly tunable: the spectral position of the resonance peak is sensitive to factors such as nanoparticle material, size, geometry, and the refractive index of the surrounding medium. Unlike propagating surface plasmons, which require specific coupling conditions at smooth metal–dielectric interfaces, LSPR is supported directly by finite-sized nanoparticles. Its oscillations are confined to the nanoscale and are non-propagating. For extremely small particles (<10 nm), classical continuum models require quantum corrections, such as incorporating enhanced electron surface scattering that leads to resonance broadening. In summary, LSPR efficiently converts light energy into localized electron oscillations and near-field enhancements at the nanoscale, forming a fundamental physical basis for applications including surface-enhanced spectroscopy, highly sensitive sensing, nanocatalysis, and photothermal therapy.

**Figure 1:**
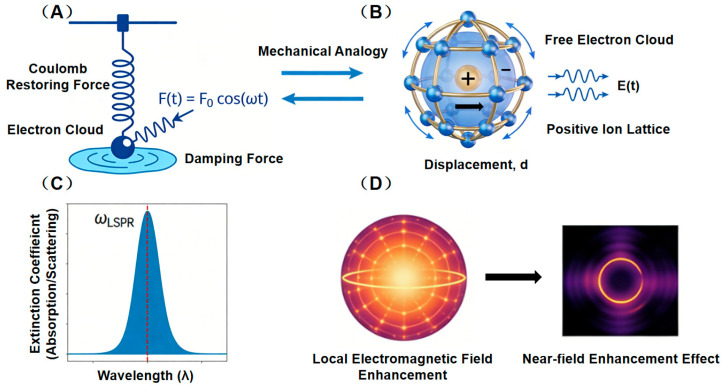
Classical Driven-Damped Oscillator Model for LSPR. (**A**) Driven-Damped Harmonic Oscillator System. (**B**) Physical Picture of a Metallic Nanoparticle. (**C**) Resonance Spectrum. (**D**) Localized Electromagnetic Field Enhancement.

A schematic of the classical driven-damped oscillator model for LSPR is shown in Figure 1. Panel (A) illustrates a driven damped harmonic oscillator system, where the periodic external force F(t)=Fcos(ωt) represents the driving effect of the alternating electric field of the incident light. The spring restoring force corresponds to the Coulomb restoring force induced by charge separation between the electron cloud and the positive ion lattice, while viscous damping simulates energy dissipation due to electron scattering. Panel (B) depicts the physical reality in a metallic nanoparticle: under the driving electric field E(t), the free electron cloud collectively shifts relative to the lattice by a displacement d, with dynamics exactly analogous to the harmonic oscillator. Panels (C) and (D) present two key outcomes of resonance: the extinction spectrum exhibits a characteristic Lorentzian peak centered at ωLSPR, and the near-field distribution reveals a strongly enhanced and highly localized electromagnetic field, especially at the nanoparticle poles. This model visually elucidates that resonance occurs when the light frequency matches the natural frequency of the collective electron oscillations, leading to a dramatic increase in the optical absorption/scattering cross-section of the nanostructure and generating intense localized field enhancement—forming the core physical principle for applications such as surface-enhanced spectroscopy.

**Figure 2:**
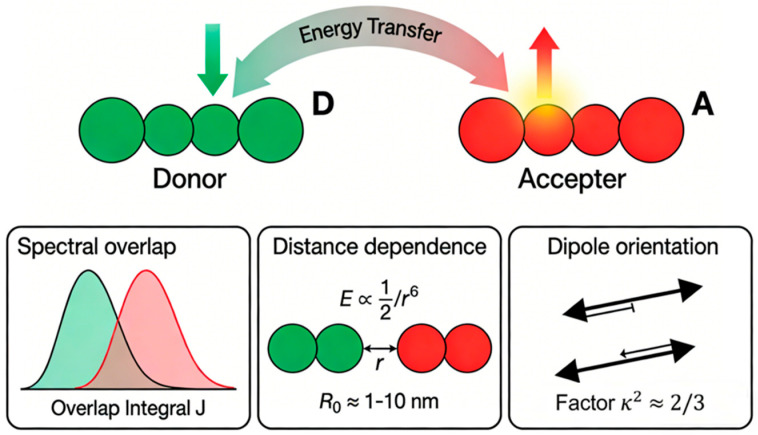
Schematic Illustration of the FRET Process.

Förster resonance energy transfer (FRET), first proposed by Theodor Förster in 1948, represents a non-radiative energy transfer mechanism mediated through dipole–dipole coupling between molecular entities. As illustrated in Figure 2, this process involves an excited-state donor molecule (e.g., a fluorophore) transferring energy to a proximal acceptor molecule, resulting in significant quenching of donor fluorescence emission. Depending on the acceptor’s intrinsic properties, this may lead to either enhanced fluorescence (sensitized emission) or quenching phenomena. The critical parameters governing this technique include: (1) Substantial spectral overlap between donor emission and acceptor absorption spectra (with transfer efficiency positively correlated to the overlap integral J value). (2) An intermolecular separation within the Förster radius (R0) range of 1–10 nm, where efficiency exhibits an inverse sixth-power dependence on distance. (3) Favorable spatial orientation of donor and acceptor transition dipole moments.

Based on the aforementioned principles of LSPR enhancement and FRET modulation, combined with our investigation and analysis of the properties of existing GNCs and QDs, we successfully designed and fabricated the GNCs-PQDs composite nanomaterials and their corresponding fluorescent probe, MB-GNCs-PQDs. In the following sections, a detailed analysis will be conducted on the characterization results of their structure, optical properties, and sensing performance based on the above theoretical basis.

### 3.2. Transmission Electron Microscopy (TEM) Analysis

**Figure 3:**
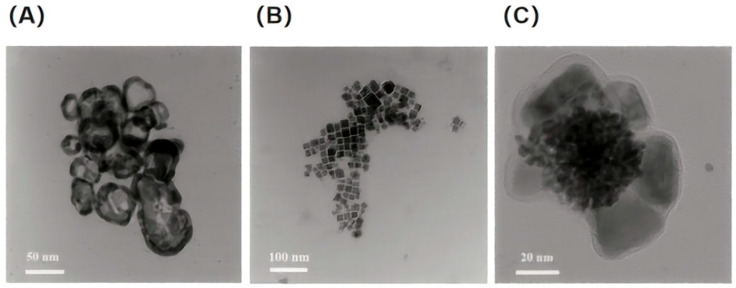
(**A**) TEM image of gold nanocages. (**B**) TEM image of perovskite quantum dots. (**C**) TEM image of the gold nanocage and perovskite quantum dot composite material.

The transmission electron microscopy (TEM) image of gold nanocages (GNCs) is depicted in Figure 3A. It can be clearly observed that the GNC particles exhibit uniform morphology with a typical regular hollow ring structure. This TEM contrast characteristic of a bright center and a dark edge serves as a definitive criterion for the hollow structure of GNCs, which has been extensively reported and validated in numerous classic publications in the field of nanomaterials. Based on the analysis of the structural characterization features of gold nanocages elaborated in the review by Xia et al. in Chemical Reviews [21], it can be concluded that the dark annular band at the outer contour corresponds to the gold shell, while the light-colored central region represents the hollow inner cavity, which is in perfect agreement with the designed synthetic target. Further analysis indicates that the GNCs prepared in this experiment possess a uniform shell thickness, with only slight roughness at the edges. They exhibit excellent overall structural integrity without any obvious cracking or collapse. The diameter distribution of individual particles is narrow, with an average size of approximately 50 nm.

The TEM image of perovskite quantum dots (PQDs) is presented in Figure 3B. When the temperature was increased to 160 °C, the synthesized PQDs underwent rapid growth within a short period. Their average particle size reached nearly 18 nm, accompanied by an increase in particle size, a narrower size distribution, more regular morphology, and excellent monodispersity. The TEM image of the GNC-PQD composite material is shown in Figure 3C. The PQDs display a close-packed morphology and are confined within the cavities of GNCs via the amide reaction. The GNCs encapsulate the PQDs, forming an outer shell with a thickness of approximately 20 nm.

### 3.3. X-Ray Diffraction (XRD) Pattern Analysis

**Figure 4:**
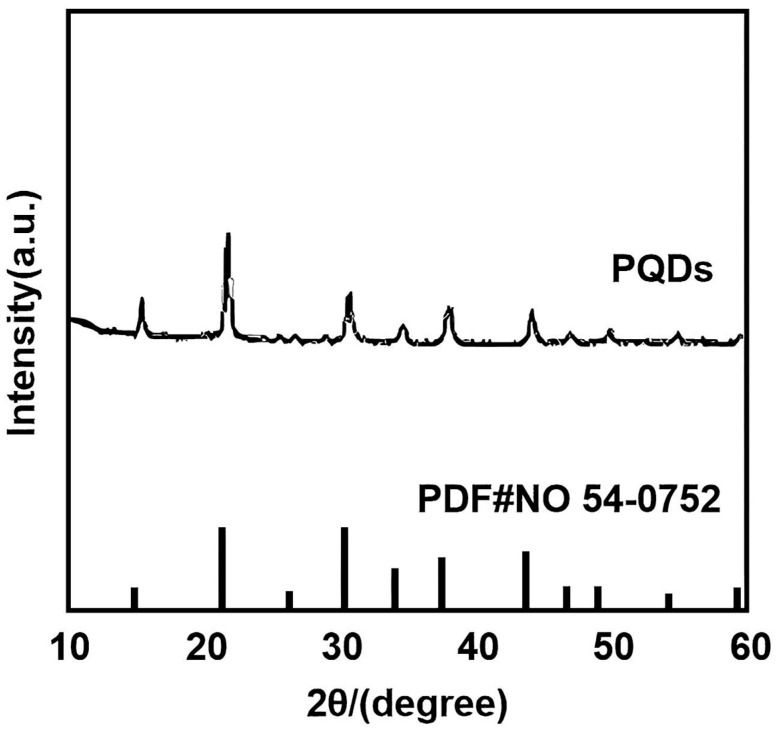
X-ray diffraction pattern of perovskite quantum dots.

The XRD pattern of the perovskite quantum dots is illustrated in Figure 4. Comparative analysis with the standard reference card of CsPbBr_3_ (JCPDS No. 54-0752) demonstrates that the diffraction peaks of the synthesized quantum dots exhibit excellent alignment with the standard peak positions, with no observable impurity peaks detected [22].

### 3.4. Fluorescence Lifetime Analysis

**Figure 5:**
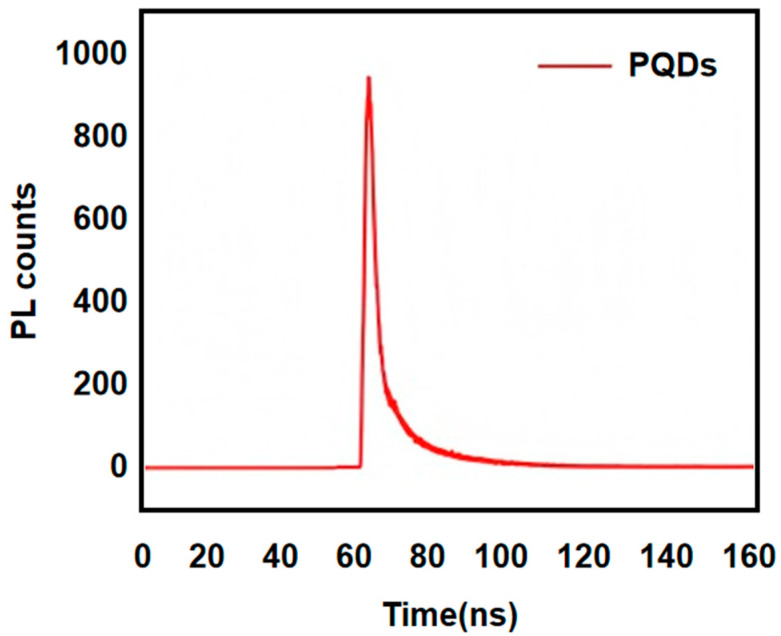
Fluorescence lifetime mapping of perovskite quantum dots.

The fluorescence lifetime characterization curve of PQDs, as illustrated in Figure 5, demonstrates that within the 0–50 ns interval, the PL counts remain near baseline levels, corresponding to the excitation source pulse duration period where carriers undergo energy relaxation without significant radiative transitions. At approximately 50 ns, the PL counts abruptly surge to a peak value nearing 900, indicating that excited-state carriers predominantly undergo band-edge radiative recombination as the dominant fluorescence emission mechanism after completing energy relaxation. Subsequently, the curve exhibits rapid decay and approaches zero beyond 100 ns, reflecting the cooperative de-excitation process of carriers through both radiative and non-radiative transitions.

### 3.5. Photothermal Response Analysis

**Figure 6:**
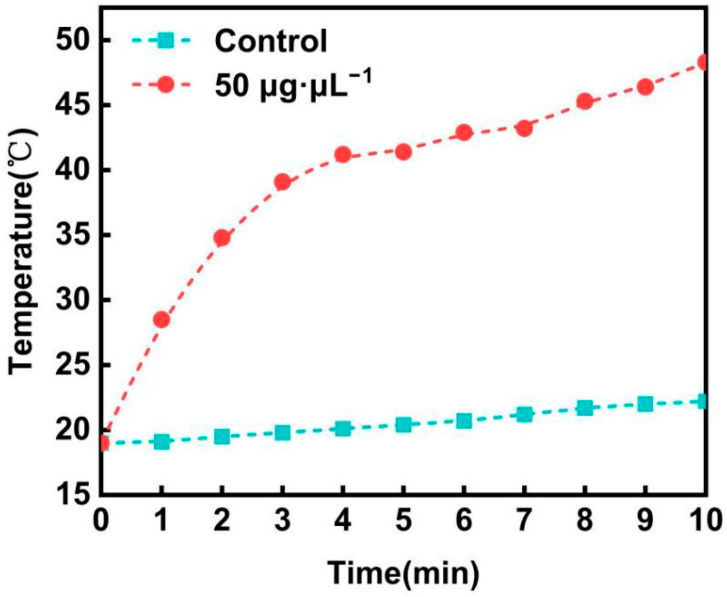
Photothermal response temperature-time profile of gold nanocages.

The photothermal conversion performance of GNCs under continuous irradiation with a 1.5 W infrared laser (wavelength: 808 nm) is illustrated in Figure 6. A significant difference in temperature response was observed between the experimental group (50 μg·μL^−1^ GNC solution) and the control group (pure water). In the experimental group, the temperature increased rapidly in the first 120 s, followed by a sustained slow rise, reaching 47 °C at 660 s with a cumulative temperature increase of 30 °C. This remarkable photothermal effect originated from the combined action of the LSPR effect and cavity thermal convection, whereas the pure water group exhibited negligible temperature fluctuation. Notably, the heating rate of GNCs decreased after 120 s, which was mainly attributed to the system approaching a state of thermal equilibrium. In the initial stage, under 808 nm laser irradiation, GNCs efficiently absorbed light energy and converted it into thermal energy via the LSPR effect. During this period, the heat generation rate was higher than the heat dissipation rate, leading to a rapid temperature increase. As time progressed, the bulk temperature of the solution increased, resulting in a larger temperature difference between the solution and the surrounding environment (air and container wall). Consequently, the rate of heat dissipation caused by thermal conduction and convection increased accordingly. When the heat generation rate and heat dissipation rate tended to dynamic equilibrium, the slope of the temperature-time curve decreased, presenting a slow, nearly linear increasing trend until a stable plateau temperature was achieved.

To clarify the advantages of the photothermal performance of the GNCs prepared in this work, their photothermal conversion efficiency (η) was calculated from the slope of the temperature rise curve, which ranged from 40% to 50%. As a classic material in the field of photothermal research, the photothermal conversion efficiency of gold nanorods (Au NRs) has been extensively investigated and reported. For instance, a study published by von Maltzahn et al. in Cancer Research indicated that its efficiency is approximately 22%, a result that has gained high recognition in the field [21]. In addition, the photothermal conversion efficiency of traditional Au NRs generally falls within the range of 20% to 30%. In comparison, the photothermal conversion efficiency of the GNCs prepared in this work is significantly higher than the typical performance level of Au NRs. This advantage endows the GNCs with important practical value in photothermal application scenarios that require the maintenance of a high-temperature steady state, and also provides crucial performance support for the subsequent translation of GNCs into practical applications.

### 3.6. Absorption Spectroanalysis

**Figure 7:**
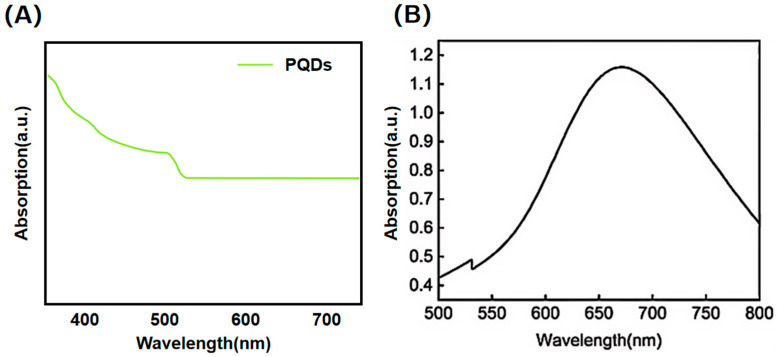
(**A**) Absorption spectrum of perovskite quantum dots. (**B**) Absorption spectrum of gold nanocages.

The absorption spectrum of perovskite quantum dots (PQDs) is illustrated in Figure 7A. Within the wavelength range of 400–700 nm, the absorbance of PQDs exhibits an overall decreasing trend with increasing wavelength. A relatively high absorbance is observed near 400 nm, which gradually declines thereafter. A distinct inflection point appears around 500 nm, beyond which the absorbance stabilizes. This absorption profile reflects the wavelength-dependent light absorption capability of PQDs, demonstrating stronger absorption for shorter wavelengths that progressively weakens and stabilizes with increasing wavelength, thereby revealing the unique photonic absorption behavior characteristic of perovskite quantum dots.

The absorption spectrum of gold nanocages (GNCs) with approximately 50 nm dimensions demonstrates their optical response characteristics across specific wavelength ranges (Figure 7B). The absorption curve displays a prominent peak near 660 nm, reaching a maximum absorbance of 1.15. This characteristic peak originates from the localized surface plasmon resonance (LSPR) effect inherent to GNCs. When the incident light wavelength matches the collective oscillation frequency of surface free electrons in gold nanocages, efficient energy absorption occurs through resonant coupling, resulting in this intensity maximum at the specific wavelength. Compared to conventional solid gold nanoparticles, the hollow porous structure of nanocages induces a significant red-shift in the LSPR peak into the near-infrared region, attributable to reduced geometric symmetry and the presence of internal cavities.

### 3.7. Fluorescence Spectroscopy Analysis

**Figure 8:**
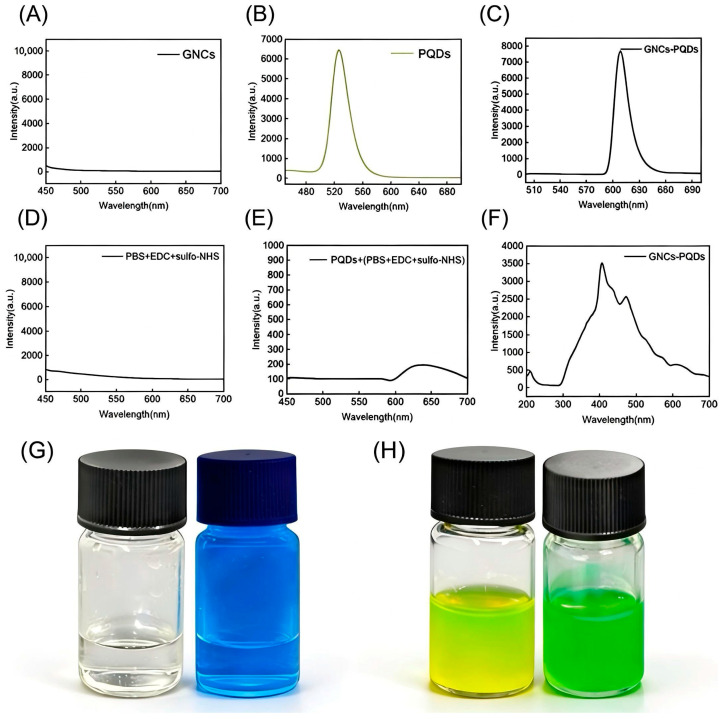
(**A**) Fluorescence spectrum of gold nanocages. (**B**) Fluorescence spectrum of perovskite quantum dots. (**C**) Fluorescence spectrum of gold nanocages and perovskite quantum dots. (**D**) Fluorescence spectrum of chemical reagents. (**E**) Fluorescence spectrum of perovskite quantum dots and chemical reagents. (**F**) Fluorescence spectrum of direct contact between gold nanocages and perovskite quantum dots. (**G**) Photographs of gold nanocages and perovskite quantum dots under natural light (appears white) and ultraviolet flashlight (365 nm, flashlight). (**H**) Photographs of perovskite quantum dots under natural light (appears yellow) and ultraviolet UV flashlight (365 nm, appears green).

The fluorescence spectrum of GNCs is depicted in Figure 8A. In the wavelength range of 450–700 nm, their fluorescence intensity remained consistently below 500 arbitrary units (a.u.), with a flat curve lacking characteristic peaks. This signal originated from the dark current/background noise of the instrument, confirming that GNCs themselves are not fluorescent emitters and thus will not introduce background interference to the fluorescence signal of the subsequent composite system. The fluorescence spectrum of PQDs is presented in Figure 8B. Under excitation with 365 nm light, a distinct characteristic emission peak appeared at approximately 520 nm, indicating the maximum fluorescence emission intensity at this wavelength and reflecting a specific photoluminescent response. The peak intensity exceeded 6000 a.u., demonstrating that PQDs possess excellent fluorescence emission capability.

The luminescent properties of the gold nanocage-perovskite quantum dot composite system (GNCs-PQDs) are illustrated in Figure 8C. A strong, sharp fluorescence peak was observed in the range of 590~650 nm, with the peak intensity 7672 a.u. at 608 nm. Compared with pure PQDs, the GNCs-PQDs composite system exhibited significant luminescence enhancement and a peak red-shift phenomenon. The luminescence enhancement was attributed to the localized surface plasmon resonance (LSPR) effect of GNCs: on the one hand, a strong local electromagnetic field was formed to improve the exciton generation efficiency of PQDs; on the other hand, interfacial charge transfer passivated the surface defects of PQDs and suppressed non-radiative recombination. Meanwhile, plasmon-exciton coupling accelerated the radiative recombination rate. Ultimately, a 15.38% increase in fluorescence intensity was achieved, which is the average value of three repeated preparations and experiments. The peak red-shift was caused by two factors: first, the strong LSPR electromagnetic field polarized the electron cloud of PQDs, reconstructing the energy levels and reducing the band gap; second, the charge transfer and lattice stress between GNCs and PQDs further narrowed the band gap, leading to a red-shift in the emission wavelength to 590~650 nm, without compromising the narrow-bandwidth luminescent property of PQDs.

Figure 8D shows the fluorescence spectrum of the chemical reagent used in the composite process of GNCs and PQDs. The intensity was extremely low across the entire wavelength range with no characteristic peaks, indicating that the reagent itself does not generate fluorescence signals, thus excluding its direct contribution to the luminescence of the composite system. The flat intensity curve also indicates that the reagent has no selective absorption or scattering effect in the visible light region, and its role is limited to chemical modification. Figure 8E presents the spectrum of the composite system of PQDs and the aforementioned chemical reagent. Although its emission peak covers the range of 450~700 nm, the overall intensity is relatively low, with only a slight increase near 620 nm, and a significant red-shift occurred compared with the emission peak of pure PQDs. This phenomenon may be attributed to the chemical modification of the surface ligands of PQDs by the reagent, which not only fails to effectively improve the fluorescence performance but may even increase non-radiative recombination due to the destruction of the ligand structure. This further highlights the positive regulatory advantage of GNCs on the fluorescence of PQDs. Figure 8F displays the fluorescence spectrum of the direct contact system of GNCs and PQDs. After the composite of PQDs (originally with an emission peak at 520 nm) with GNCs, the intrinsic fluorescence peak at 520 nm was retained, and a new significantly enhanced fluorescence peak appeared at approximately 420 nm. This further confirms that the LSPR effect of GNCs can flexibly regulate the fluorescence properties of PQDs, providing more possibilities for the spectral optimization of composite systems. Figure 8G shows the actual photographs of the GNCs-PQDs composite system under natural light and 365 nm ultraviolet (UV) flashlight. Under UV irradiation, both the bottle and the GNCs-PQDs reagent exhibited a blue color, indicating that the blue origin is the UV flashlight, which may be attributed to its relatively broad emission band. No obvious difference was observed, indicating that the composite process and light conditions did not damage the macroscopic stability of the material. Figure 8H shows the actual photograph of the PQD solution under 365 nm UV light, which exhibits bright green fluorescence. This is in perfect correspondence with the spectral peak characteristic at 520 nm in Figure 8B, further verifying the excellent fluorescence performance of PQDs from the macroscopic visual perspective.

## 4. Application Detection

MicroRNAs (miRNAs) play a pivotal role in tumorigenesis and cancer progression, among which miRNA-4529-3P and miR-301b-3p demonstrate significant potential as early diagnostic biomarkers for non-small cell lung cancer (NSCLC), accounting for approximately 80% of all lung cancer cases. However, due to the small molecular size and low abundance of these miRNAs in body fluids, they impose stringent requirements on detection probes [23,24,25,26]. Current mainstream NSCLC detection methodologies exhibit notable limitations, including insufficient specificity, high costs for dynamic monitoring, and inconsistent results, thereby creating an urgent need for highly sensitive, safe, and efficient diagnostic approaches.

Fluorescent probe detection technology has emerged as a promising solution for tumor detection owing to its superior selectivity, high sensitivity, rapid response, operational feasibility, and excellent biocompatibility. This technique employs specifically designed fluorescent probes that bind to target molecules, enabling precise detection. For NSCLC-specific miRNAs, custom-designed fluorescent probes can be synthesized based on complementary DNA strands and miRNA base sequences, offering an effective strategy to overcome the shortcomings of existing detection methods.

**Figure 9:**
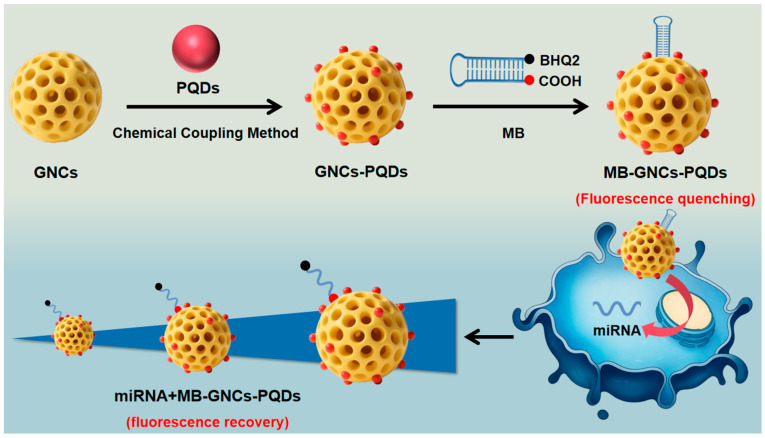
Schematic illustration of the preparation process for miRNA-conjugated magnetic bead-graphene quantum dot nanocomposites (miRNA + MB-GNCs-PQDs).

For experimental procedures, 100 μL of the prepared MB-GNCs-PQDs fluorescent probe solution was aliquoted for subsequent use. Standard solutions of biological miRNA-4529-3P and miR-301b-3p were prepared at appropriate concentrations, with 20 μL aliquots mixed thoroughly with the probe solution using a vortex mixer. Following 40 min of incubation at room temperature to complete the reaction, the entire synthesis protocol was executed as illustrated in Figure 9. miRNA was synthesized and purified by Shanghai Sangon Biotech Co., Ltd. (Shanghai, China). The base sequences required for the preparation of the fluorescent probe MB-GNCs-PQDs and the detection of miRNA are shown in Table 1.

**Figure 10:**
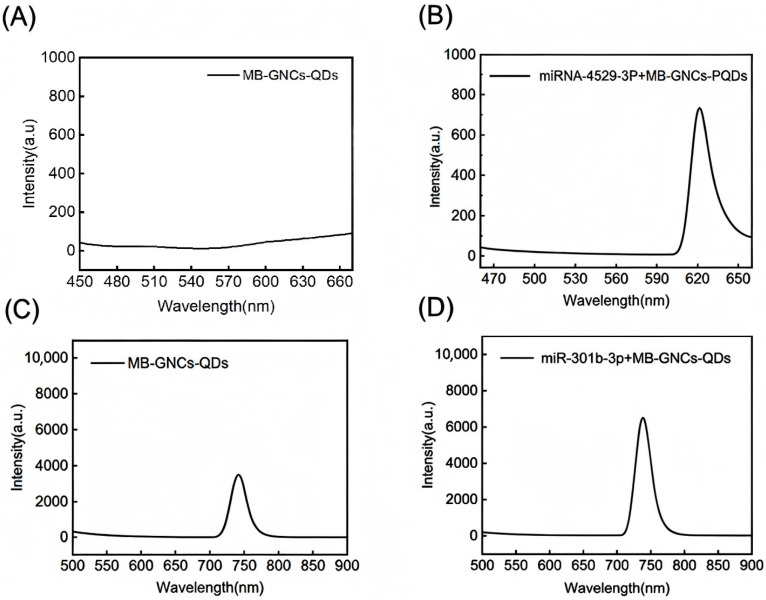
(**A**) Fluorescence spectrum of MB-GNCs-PQDs. (**B**) Fluorescence spectrum of miRNA-4529-3P + MB-GNCs-PQDs. (**C**) Fluorescence spectrum of MB-GNCs-PQDs. (**D**) Fluorescence spectrum of miR-301b-3p + MB-GNCs-PQDs.

Figure 10A displays the fluorescence spectrum of the MB-GNCs-PQDs probe constructed with molecular beacons (MB), 50 nm gold nanocages (GNCs), and perovskite quantum dots (PQDs), using BHQ2 as the quencher. BHQ2 is a small-molecule quencher with an efficient absorption band ranging from 550 to 650 nm [27]. As shown in Figure 9, MB exists in a stem-loop closed conformation, with one end modified with PQDs (the fluorescent donor) and the other end modified with BHQ2. The complementary base pairing in the stem region causes the long base chain to fold (close), resulting in an extremely short spatial distance between PQDs and BHQ2. The stem region of the molecular beacon (MB) contains approximately 5~7 base pairs, with each base pair being about 0.34 nm in length. Together with the length of the linker arm, it can be estimated that the spatial distance between PQDs and BHQ2 in the “closed” state falls within the range of 3~5 nm, which satisfies the spatial requirement for efficient FRET quenching (1~10 nm) mentioned in Section 3.1. After the construction of the fluorescent probe, the fluorescence intensity at 608 nm decreased from 7672 a.u. to 50.65 a.u., and the quenching efficiency can be calculated to be nearly 99.35%. The probe exhibits negligible fluorescence intensity within the 500~700 nm wavelength range due to two primary mechanisms: (1) In Förster resonance energy transfer (FRET), the absorption spectrum of BHQ2 precisely overlaps with the emission spectrum of PQDs. Upon excitation, PQDs in the high-energy state transfer energy to BHQ2 non-radiatively, which subsequently dissipates as thermal energy rather than photon emission. (2) In direct electron transfer, the energy level difference between PQDs and BHQ2 enables excited-state electrons from the conduction band of PQDs to directly transfer to lower-energy orbitals of BHQ2 at close range, disrupting the exciton equilibrium (electron–hole pair separation) in PQDs and completely suppressing radiative recombination. The synergistic effect of these dual mechanisms results in “complete quenching” of PQD’s fluorescence.

Figure 10B illustrates the fluorescence spectral characteristics of the miRNA-4529-3P/MB-GNCs-PQDs complex. While the peak positions remain largely unchanged compared to MB-GNCs-PQDs alone, the fluorescence intensity increases to 700 a.u. Upon hybridization with miRNA-4529-3P, the target sequence forms specific base pairs with the loop region of MB, stabilizing a duplex structure that forces the stem-loop to unfold and extend. This conformational change significantly increases the spatial separation between PQDs and BHQ2, disrupting the quenching pathway: The enlarged distance drastically reduces FRET efficiency, preventing energy transfer from excited PQDs to BHQ2, thereby promoting photon emission. Concurrently, the lengthened electron-transfer distance between PQDs and BHQ2 halts non-radiative decay, allowing excitons to return to the ground state via radiative recombination and emit fluorescence.

Figure 10C presents the fluorescence spectrum of MB-GNCs-PQDs probes fabricated with 100 nm GNCs. Similarly, the 500–700 nm range shows minimal fluorescence, while a distinct peak emerges at 740 nm, followed by gradual intensity decay at longer wavelengths. This phenomenon arises because PQDs possess inherent emission wavelengths; when excited beyond BHQ2’s effective quenching range and outside the localized surface plasmon resonance (LSPR) absorption band of GNCs, their fluorescence dominates, generating the characteristic peak.

Figure 10D depicts the fluorescence spectrum of MB-GNCs-PQDs upon recognizing miR-301b-3p, exhibiting a sharp emission peak exceeding 6000 a.u. in the 700–750 nm band. Target binding induces MB unfolding, distancing BHQ2 and nullifying its quenching effect on GNCs-PQDs. Consequently, the intrinsic luminescence of GNCs-PQDs is fully unleashed, potentially amplified by GNCs’ enhancement effects to produce the ultra-intense sharp peak.

Notably, the combination of GNCs-PQDs outperforms traditional fluorescent probes in miRNA detection. The core advantage stems from the triple synergistic effect generated by the composite system constructed by GNCs, PQDs, and molecular beacons (MB). This system realizes the integrated integration of a signal enhancement unit (GNCs), a high-efficiency signal output unit (PQDs), and an intelligent recognition switch (MB), exhibiting more prominent comprehensive advantages compared with single-component or simply functionalized probes. Mechanistically, the aforementioned LSPR-mediated fluorescence enhancement effect is the core manifestation of the synergistic effect, providing a sufficient signal basis for the detection of low-concentration targets. Meanwhile, the advantages of background suppression and high signal-to-noise ratio (SNR) are achieved through the FRET mechanism. MB precisely localizes the quencher group BHQ2 in the vicinity of PQDs, rendering the probe in a “closed” state with extremely low background in the absence of targets. The target-triggered conformational change in MB can efficiently relieve the quenching, forming a huge signal on-off ratio to ensure detection specificity. In addition, the inherent excellent photothermal conversion capability of GNCs is completely retained in the composite system, enabling this probe to potentially develop into a theranostic integrated platform with both miRNA fluorescence detection and photothermal therapy functions in the future, significantly expanding its application scenarios.

## 5. Conclusions

This study successfully designed and fabricated a novel fluorescent probe based on gold nanocage-composited perovskite quantum dots. Through rational surface modification and composite structure design, the advantages of gold nanocages (GNCs) and perovskite quantum dots were fully exploited, and the enhancement of fluorescence performance was achieved. Studies on its luminescent properties revealed that the GNCs-PQDs composite nanomaterial exhibited a new emission peak in the wavelength range of 600–650 nm, with a fluorescence enhancement of approximately 15.38% compared with pure PQDs. Subsequently, molecular beacons (MBs) conjugated with the quencher group BHQ2 were immobilized on the surface of GNCs-PQDs via the amide reaction, thus completing the preparation of the fluorescent probe MB-GNCs-PQDs. When this probe was used to separately detect miRNA-4529-3P and miR-301b-3p—two specific biomarkers for lung cancer—the results demonstrated that it could effectively recognize the corresponding tumor markers.

The current work has focused on the preparation of the probe. Given its promising biomedical application prospects, the necessary steps for subsequent research include systematic in vitro cytotoxicity evaluation, in vivo distribution and metabolism studies, and biosafety assessments. These studies are crucial to promote the translation of this probe into practical clinical diagnosis or theranostic integrated applications.

## Figures and Tables

**Table 1:** Nucleotide sequences of DNA strands and miRNAs.

Name	Sequences (5′-3′)
miRNA-4529-3P	AUUGGACUGCUGAUGGCCCGU
MB-miRNA-4529-3P	COOH-ACGGGCCAUCAGCAGUCCA-BHQ2
miR-301b-3p	CAGUGCAAUGAUAUUGUCAAAGC
MB-miR-301b-3p	COOH-GCUUUGACAAUAGUAUUGCACUG-BHQ2

## Data Availability

Data will be made available upon request.
